# Responses of persons at risk of suicide: A critical interpretive synthesis

**DOI:** 10.1002/nop2.169

**Published:** 2018-07-10

**Authors:** Anne‐Grethe Talseth, Fredricka L. Gilje

**Affiliations:** ^1^ Faculty of Health Sciences, Department of Health and Care Sciences University of Tromsø, The Arctic University of Norway Tromsø Norway; ^2^ College of Nursing Montana State University Billings Montana

**Keywords:** critical interpretive synthesis, nursing, patients, qualitative content analysis, suicide

## Abstract

**Aim:**

Several nursing studies focus on suicidal persons; yet, a synthesis of such research is unavailable. The aim of this review was to give an inclusive understanding of responses of persons at risk for suicide that guides clinical nursing practice and research.

**Design:**

A reflexive and iterative study design was used in this study.

**Method:**

A qualitative content analysis and a systematic review of literature guided the six‐phase Critical Interpretive Synthesis . A sample consisting of 24 nursing studies published during 1994–2017 were included in this study.

**Results:**

Key concepts found were “Disengaged while fraught with affliction”; “Readiness to engage in life”; and “Engaging through caring and confirming humanity.” Contextually, there are gaps in global nursing knowledge. Conceptually, three key concepts can guide the nursing practice and give an impetus for the research. Methodologically, the Critical Interpretive Synthesis served as a helpful way to summarize and synthesize a small sample size into an aggregate body of knowledge. An evidenced‐based understanding of responses of persons at risk for suicide can guide nurses to ensure safety, promote hopeful recovery, and foster resilience.

## 1 INTRODUCTION

The worldwide statistics estimate that millions of people risk suicide every year (Nock & Kessler, 2006) and approximately one million commit suicide annually (World Health Organization, 2016); yet, most survive an attempt and go on to experience living without future attempts (Owens, Horrocks, & House, 2002). Persons at risk for suicide, a very vulnerable population, are likely to encounter nurses in various settings. Understanding persons at risk of suicide is important for nurses as they ensure safety of those at risk. The way to understand persons at risk of suicide is through their experiences of being suicidal. Evidenced‐informed and evidenced‐based knowledge can enhance nurses' understanding and facilitate pivoting at‐risk persons from focusing on death to focusing on life (c.f., Cutcliffe & Santos, 2014), thus addressing urgent global initiatives to reduce suicide deaths (e.g., World Health Organization, 2016).

Though there is a myriad of evidenced‐based knowledge about persons at risk in non‐nursing literature, nursing knowledge about this topic is sparse. Nurses' understandings about responses of persons at risk for suicide should be guided by evidenced‐informed knowledge (Cutcliffe & Santos, 2014) and evidenced‐based nursing knowledge (e.g., Talseth & Gilje, 2011).

A systematic review of qualitative healthcare research (Lakeman & Fitzgerald, 2008) published between 1997 and 2007 reported five themes describing how persons lived with or recovered from suicidal ideations. These themes were: “suffering;” “struggle;” “connection;” “turning points,”; and “suicide and coping.” A follow‐up article (Lakeman, 2010) described how these five themes informed about nursing practice and empathic care. Of note is that the inclusion criteria for the Lakeman and Fitzgerald study (2008) was not solely nursing research; 16 of the 20 articles were authored by non‐nurses. An aggregate study of evidenced‐based nursing research on this topic is unavailable in published literature. An updated inclusive view of this topic based on a synthesis of qualitative and quantitative nursing research could increase and enhance nursing's global evidenced‐based knowledge, guide nursing practice while ensuring patient safety and serve as an impetus for future nursing research.

## 2 AIM

The aim of this review was to give an inclusive understanding of responses of persons at risk for suicide based on the published nursing research. The intent was to guide nurses as they encounter persons at risk of suicide in clinical nursing practice and to inform future nursing research.

## 3 METHOD

A Critical Interpretive Synthesis (CIS) was conducted (Dixon‐Woods, et al., 2006). This six‐phase iterative, reflexive review approach consisted of formulating the review question, search the literature, sampling, determining quality, extracting data, and developing an interpretive synthesis. Such reviews guide selecting relevant, quality works from a large amount of available information and increasing clarity of current knowledge; systematic reviews can offer workable practice solutions (Sandelowski, 2008).

### 3.1 Formulating the review question

The review question was: “What is a CIS of nursing research addressing responses of persons at risk for suicide?” The aim was to increase the understanding that would benefit nursing practice and give an impetus for future research. The review question was initially apparent to the researchers because they had previously conducted a CIS on nurses' experiences with suicidal patients (Talseth & Gilje, 2011).

### 3.2 Searching the literature

The search strategy proceeded in several phases. To begin, we developed inclusion and exclusion criteria. Inclusion criteria were nursing research studies with at least one nurse as the author, published in peer‐reviewed nursing journals and peer‐reviewed healthcare journals between 1990 and 2007 in English language, and available in electronic databases. Content criteria for inclusion were studies that focused on patients' responses to being suicidal. Exclusion criteria were all studies in non‐English languages, and those classified as reviews, published in books, and dissertations.

A flow diagram, Preferred Reporting Items for Systematic Reviews and Meta‐Analysis (PRISMA) (Mohler, Liberati, Tetzlaff, & Altman; The PRISMA Group, 2009), was used to improve reporting of the systematic review. The four items in the PRIMSA flowchart are Identification, Screening, Eligibility, and Inclusion.

**Figure 1:**
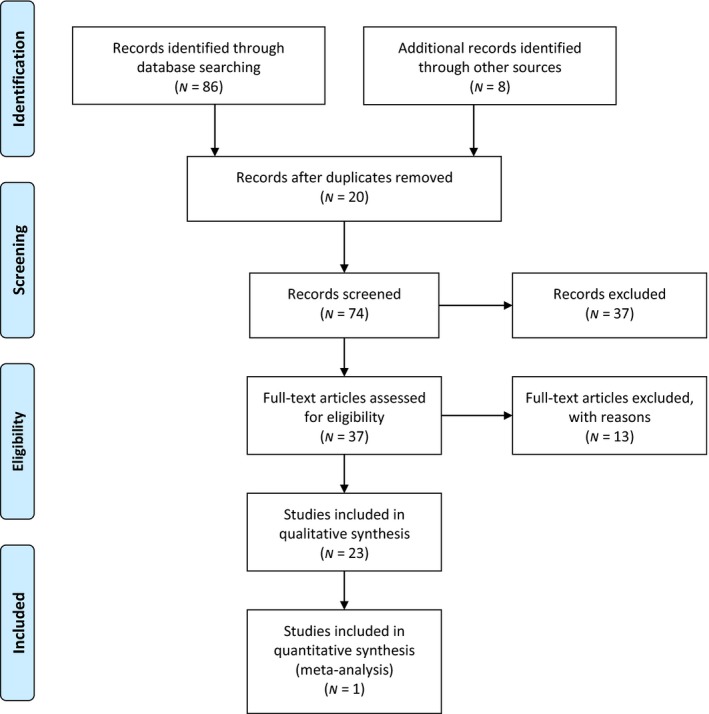
PRISMA 2009 flow diagram. Source:Mohler et al. (2009)

Identification of records occurred though searching electronic databases CINAHL Medline, Ovid, Psychinfo, and PubMed using the following search terms for each database: CIS, experiences, nursing research, patients, suicide, suicidal. These terms were searched individually and collectively resulting in 86 studies. Eight additional studies identified through other sources (i.e., reference lists of the 86 studies) were also included for screening. The screening was carried out by examining titles and abstracts of the 94 studies. A total of 20 duplicate studies were found and were removed. With further screening, 37 of the 74 studies were excluded because they did not fit the inclusion criteria. The remaining 37 full‐text studies were then assessed by both authors for eligibility based on relevancy to the aim of this study and the inclusion criteria. Of the 37 eligible studies, 13 were excluded based on sampling, authorship, secondary studies, or did not meet quality appraisal. Based on results of PRISMA, 24 nursing research studies were included in the sample (see Figure 1).

### 3.3 Sampling

From the systematic review of articles, the purpose sample comprised of 24 studies published from 1994 ‐ May 2017. While our literature search started with the publication year 1990, the first published study found was dated 1994. Our rationale for including this study in the sample was: to focus on an inclusive perspective of the research topic of interest; the study's research question and results were relevant; and the study met inclusion criteria (Table 1).

**Table 1:** Description of sample

Author(s), Year, Journal	Design Method Date Collection Data Analysis	Sample Participants Setting Location	Aim/Research Question/Hypothesis
Carrigan (1994), *Journal of Advanced Nursing*	Qualitative Exploratory Interviews Content analysis	Convenience: *N* = 6 Age: Unreported Gender: Female = 3 Male = 3 Ethnicity: Unreported Dx: History of suicide Setting: General and psychiatric Location: Ireland	Highlight psychosocial needs as perceived by individuals who survived attempted suicide through self‐poisoning
Valente (1994), *Clinical Nursing Research*	Qualitative Descriptive Thematic analysis of suicide message from clinical records Thematic analysis	Purposive: *N* = 50 Age: 19–75 years Gender: Male = 50 Ethnicity: Unreported Dx: Suicide completers and parasuicidal patients Setting: Veterans Affairs Medical Center Location: USA	Examine messages of suicide completers and compare these messages with a matched cohort of message from parasuicidal, psychiatric patients
Pitula and Cardell (1996), *Psychiatric Services*	Qualitative Descriptive Phenomenology Interview Interpretive	Convenience: *N* = 14 Age: 21–47 years Gender: Female = 8 Male = 6 Ethnicity: Unreported Dx: Suicidal Setting: Inpatient psychiatric Location: USA	Examine the experience of constant observation from the patients' point of view
Walsh and Minor‐Schork (1997), *Applied Nursing Research*	Qualitative Exploratory Interview Thematic analysis	Convenience: *N* = 11 Age: 13–18 Gender: Unreported Ethnicity: Unreported Dx: Depressed and suicidal Setting: Inpatient psychiatric Location: USA	Explore reactions of hospitalized, suicidal adult to an art future image intervention (AFI)
Moore (1997), *Archives of Psychiatric Nursing*	Qualitative Hermeneutic phenomenology Interview Interpretative	Purposive: *N* = 11 Age: 64–92 years Gender: Unreported Ethnicity: Unreported Dx: Suicidal Setting: Inpatient psychiatric Location: Canada	Explore how older adults who were suicidal experience meaning in their lives.
Haight and Hendrix (1998), *Suicide and Life‐Threatening Behavior*	Qualitative Exploratory Life Review and Experiencing Form Content analysis	Purposive: *N* = 12 Age: Average = 80 years Gender: Female = 12 Male = 0 Ethnicity: Caucasian Dx: Nonsuicidal or Suicidal Setting: Nursing home Location: USA	Explore Maris (1981) hypothesis of suicidal careers by comparing life stories of six satisfied ageing women with life stories of six women who verbalized suicide intent
Cardell and Pitula (1999), *Psychiatric Services*	Qualitative Interview Grounded Theory Interactive analyses	Purposive: *N* = 20 Age: Mean = 32 years Gender: Male = 7 Female = 13 Ethnicity: Unreported Dx: Major depression, borderline personality disorder, bipolar, schizoaffective, dysthymia, psychoses NOS, adjustment disorder with depressed mood, alcohol abuse or Schizophreniform disorder Setting: Inpatient psychiatric Location: USA	Explore patients' experiences of constant observation to determine whether they derived any therapeutic benefits beyond intended protective benefits
Talseth et al. (1999), *Journal of Advanced Nursing*	Qualitative Phenomenological Hermeneutic Interview Interpretative	Convenience: *N* = 21 Age: 25–63 years Gender: Female = 12 Male = 9 Ethnicity: Norwegian Dx: Suicidal Setting: Inpatient psychiatric Location: Norway	Illuminate the meaning of suicidal psychiatric inpatients' experiences of being cared for by mental health nurses
Samuelson et al., 2000, *Journal of Advanced Nursing*	Qualitative Descriptive Interview Content analysis	Convenience: *N* = 18 Age: 18–53 years Gender: Female = 6 Male = 12 Ethnicity: Swedish Dx: Suicidal Setting: Inpatient psychiatric Location: Sweden	Describe the attempted suicide patients' perception of receiving specialized inpatient psychiatric care
Tzeng (2001), *Journal of Transcultural Nursing*	Qualitative Hermeneutic phenomenological Interview Thematic analysis	Purposive: *N* = 10 Age: 20–53 years Gender: Female = 5 Male = 5 Ethnicity: Asian Dx: Suicidal and adjustment disorder or major depression Settings: General and psychiatric Location: Taiwan	Understand how suicidal patients experienced their lives after attempted suicide
Talseth et al. (2003), *Nursing Ethics*	Qualitative Phenomenological hermeneutic Secondary analysis of narrative interviews Interpretative	Convenience: *N* = 2 Age: Middle‐aged Gender: Not reported Ethnicity: Norwegian Dx: Suicidal Setting: Inpatient psychiatric Location: Norway	Describe a process of consolation revealed by two suicidal patients' experiences in the light of a model of consolation
Wicklander et al. (2003), *Scandinavian Journal of Caring Science*	Qualitative Descriptive Semi‐structured interviews Thematic analysis	Convenience: *N* = 18 Age: 22–53 years Gender: Male = 5 Female = 8 Ethnicity; Unreported Setting; Inpatient psychiatric Location: Sweden	Extract experiences of shame and highlight aspects of caring associated with shame in conjunction with a suicide attempt
Cutcliffe et al. (2006), *Journal of Nursing Studies*	Qualitative Grounded theory Interview Interactive analyses	Theoretical: *N* = 20 Age: Over 18 years Gender: Unreported Ethnicity: Unreported Dx: History of suicide Setting: General and psychiatric Location: UK & USA	Determine if psychiatric/mental health nurses provide meaningful caring experiences to suicidal people, and if so, how.
Biong and Ravndal (2007), *International Journal of Qualitative Studies on Health and Wellbeing*	Qualitative Phenomenological‐ hermeneutic Narrative interview Interpretive	Convenience: *N* = 4 Gender: Male = 4 Female = 0 Age: Unreported Ethnicity: Norwegian Dx: Suicidal behaviour and substance abuse Setting: Substance abuse treatment centre Location: Norway	Illuminate experiences of suicidal behaviours in young men with long term substance abuse and interpret their narratives regarding meaning.
Biong, Karlsson, and Svensson (2008), *Journal of Psychiatric and Mental Health Nursing*	Qualitative Exploratory Secondary analysis Thematic analysis	Purposive: *N* = 4 Age: 32–40 years Gender: Male = 4 Female = 0 Ethnicity: Norwegian Dx: History of suicide Setting: General and psychiatric Location: Norway	Explores and interpret men's' experience of sense of self within context of recovery from substance abuse and suicidal behaviour.
Sharaf et al. (2008), *Journal of Child and Adolescent Psychiatric Nursing*	Quantitative Cross sectional survey High School Questionnaire & Measure of Adolescent Potential for Suicide (multidimensional assessment instruments) Hierarchical multiple regression	Validated selection Model: *N* = 849 Age: 14–21 years Gender: Unreported Ethnicity: Diverse Dx: Risk of suicide Setting: High school Location: USA	Examine the moderating effect of family support on the relationship between self‐esteem and suicide risk behaviours among potential high school dropouts
Lin et al. (2009), *Journal of Clinical Nursing*	Qualitative Phenomenology Interview Interpretative	Purposive: *N* = 12 Age: Mean 33 years. Gender: Female = 12 Male = 0 Ethnicity: Asian Setting: Inpatient psychiatric Location: Taiwan	Investigate the lived experiences of brokered brides who have attempted suicide in Taiwan.
Ku et al., (2009), *Gerontologist*	Qualitative Exploratory Interviews Thematic analysis	Random Stratification: *N* = 19 Age: 65 + years Gender: Unreported Ethnicity: Asian Dx: Unreported Setting: Veterans Home Location: Taiwan	Understand suicide experiences, especially triggers, in institutionalized veterans in Taiwan.
Holm and Severinsson (2011), *International Journal of Mental Health Nursing*	Qualitative Interview Exploratory Thematic analysis	Convenience: *N* = 13 Age: 25–53 years Average: 39 years Gender: Female = 13 Men = 0 Ethnicity: Norwegian Dx: Borderline Personality Disorder Setting: General and psychiatric Location: Norway	Explore how a recovery process facilitated changes in suicidal behaviour in women with Borderline Personality Disorder.
Vatne and Nåden (2014), *Scandinavian* *Journal* *of* *Caring* *Sciences*	Qualitative Hermeneutic Interview Thematic analysis	Convenience: *N* = 10 Age: 21–45 years Gender: Female = 4 Male = 6 Ethnicity: Norwegian Dx: History of suicide Settings: Emergency and psychiatric Location: Norway	Explore experiences of persons after suicidal crises or recently completed suicide attempts
Lees et al. (2014), *International* *Journal* *of* *Mental* *Health*	Qualitative Descriptive Interview and survey Critical discourse, constant comparison, classical content analysis and Descriptive statistics	Convenience: *N* = 9 Gender: Unreported Ethnicity: Unreported Dx: History of suicide Setting: Inpatient psychiatric Location: Australia	Explore experiences and needs that mental health consumers had of suicide crises, the role of the mental health nurse, and key factors suggested to impact quality of care
Vatne and Nåden (2014) N*ursing* *Ethics*	Qualitative Hermeneutic Interview Thematic analysis	Convenience: *N* = 10 Age: 21–52 years Gender: Male = 6 Female = 4 Ethnicity: Unreported Dx: Seriously suicidal Setting: Unreported Location: Sweden	Explore experiences of being suicidal and the encounter with healthcare personnel
Vatne and Nåden (2016a), *Nursing Ethics*	Qualitative Hermeneutic Interview Thematic analysis	Convenience: *N* = 10 Ages: 21–52 years Gender: Male = 6 Female = 4 Ethnicity: Unreported Dx: Suicidal Setting: Emergency Psychiatric Location: Sweden	What resources in the person him/her self and their surroundings that are crucial in a suicidal crisis to maintaining the will to live and hope for life
Vatne and Nåden (2016b), *Nursing Ethics*	Qualitative Hermeneutics Interview Thematic analysis	Convenience: *N* = 10 Ages: 21–52 years Gender: Male = 6 Female = 4 Ethnicity: Unreported Dx: Suicidal Setting: Emergency Psychiatric Location: Sweden	What do suicidal patients see as meaningful help in care and treatment situations?

Sixteen studies were published in peer‐reviewed nursing journals. Four were published in refereed health‐related journals (i.e., *Gerontologist, Psychiatric Services, Suicide and Life‐Threatening Behavior*and *International Journal of Mental Health*). Publication dates ranged from 1994 to May 2017; most were published since 2000.

Most study designs were qualitative (*N = *23) and published after 1994. The only quantitative (*N* = 1) study was published prior to 2008. Most studies were reportedly situated in Europe and North America, and a few in Asia, United Kingdom, and Australia. Inpatient psychiatry was the most common setting; only some were in general and psychiatric settings. Other settings included emergency and psychiatric, nursing home, veterans' home, substance abuse treatment, and education. One study location was unreported (Table 2).

**Table 2:** Sample characteristics

Characteristic	Characteristics of the Sample	*N*
Publication Sources	Refereed nursing journals	20
	Refereed medical‐psychiatric‐mental health journals (i.e., Suicide And Life‐Threatening Behavior, Gerontologist, Psychiatric Services, International Journal Of Mental Health)	4
Designs	Qualitative: 23	23
	Exploratory = 7	7
	Descriptive = 4	4
	Hermeneutic = 4	4
	Hermeneutic phenomenology = 3	3
	Phenomenology hermeneutic = 2	2
	Phenomenology = 2	2
	Grounded Theory = 1	1
	Quantitative: 1	1
*Data Analysis Methods*	Qualitative studies	
	Thematic analysis	11
	Interpretive	6
	Content analysis	3
	Interactive	3
	Quantitative studies	
	Descriptive and analytical statistics	1
*Settings*	Inpatient psychiatric	10
	General and psychiatric	5
	Emergency psychiatry	3
	Veterans home	2
	Nursing home	1
	Substance abuse treatment	1
	Education	1
	Unreported	1
*Locations*	Europe	11
	North America	7
	United Kingdom	2
	Asia	3
	Australia	1

The sample participants totalled 1,273. The majority (*N* = 849) of the participants were high school students; the remainder were mostly psychiatric patients. More than half (*N* = 18) of the study was conducted in psychiatric settings (inpatient, general and psychiatric, and emergency and psychiatric; Table 3).

**Table 3:** Sample settings, participants and designs

Setting	*N* Participants	Design	*N* Studies
Education	849	Quantitative	1
Inpatient psychiatric	136	Qualitative	10
Veterans' Home	69	Qualitative	2
General and psychiatric	53	Qualitative	5
Emergency and psychiatric	30	Qualitative	3
Unreported	20	Qualitative	1
Nursing Home	12	Qualitative	1
Substance abuse treatment	4	Qualitative	1
Total	1,273		24

### 3.4 Determining quality

All studies met the inclusion criteria. Eighteen were published in nursing journals and two in medical‐psychiatric journals. The one quantitative study was evaluated using the Jadad scale (Jadad, et al., 1996); the 23 qualitative studies were evaluated using a Critical Appraisal Skills Program (CASP) (2006).

The Jadad scale was developed to evaluate the quality of reports of randomized trials. It is used in meta‐analysis and systematic reviews. It involves a three‐point questionnaire ranging from 0 to 3 assessing randomization, blinding, and withdrawals/dropouts. Points are added if randomization and blinding are appropriately described. Critics of the scale have identified 10 flaws (Berger, 2006), noting it overemphasizes blinding and that inter‐rater reliability needs to be further evaluated (Clark, Huët, Salmi, & Laupacis, 1999). The authors agreed on including the Jaded scores which ranged from 1‐3 points.

The CASP (2006) is a 10‐item tool addressing the aim, method and design, sampling, data collection and analysis, ethical issues, validity, and relevance of results in qualitative studies. This tool calls for the rating of each of the 10 questions as “yes,” “no,” or “can't tell.” The authors chose to rate responses as “yes” or “no” because they found that these were the most helpful responses. Of the 10 questions, 9 were rated as “yes” responses in all studies. The question about ethical issues lacked the most positive responses. This question includes explaining the research to participants, adequately considering the researcher–participant relationship, discussing informed consent and confidentiality, and whether ethical committee approval had been sought. Eight of the studies had a CASP score of at least 8/10 because they did not report ethical standards. Reporting ethical standards is of concern because of its importance in all research (Lakeman & Fitzgerald, 2009) and most qualitative research views participants as co‐researchers (Table 4).

**Table 4:** Quality determinants of the sample

Studies	Source	Quality determinant
Quantitative (3/3 possible)	Sharaf et al. (2008)	Jadad = 1/3
Qualitative (10/10 possible)	Carrigan (1994)	CASP = 10/10
	Valente (1994)	CASP = 8/10
	Pitula and Cardell (1996)	CASP = 8/10
	Walsh and Minor‐Schork (1997)	CASP = 9/10
	Moore (1997)	CASP = 9/10
	Haight and Hendrix (1998)	CASP = 8/10
	Cardell and Pitula (1999)	CASP = 8/10
	Talseth et al. (1999)	CASP = 10/10
	Samuelson et al., 2000	CASP = 10/10
	Tzeng (2001)	CASP = 9/10
	Talseth et al. (2003)	CASP = 10/10
	Wicklander et al. (2003)	CASP = 10/10
	Cutcliffe et al. (2006)	CASP = 10/10
	Biong and Ravndal (2007)	CASP = 10/10
	Biong et al. (2008)	CASP = 9/10
	Lin et al. (2009)	CASP = 10/10
	Ku et al. (2009)	CASP = 10/10
	Holm and Severinsson (2011)	CASP = 10/10
	Vatne and Nåden (2014)	CASP = 10/10
	Lees et al. (2014)	CASP = 10/10
	Vatne and Nåden (2014)	CASP = 10/10
	Vatne and Nåden (2016b)	CASP = 10/10
	Vatne and Nåden (2016a)	CASP = 10/10
	

As we examined the quality determinants, contemplated their variability and considered the historical evolution of nursing research, we concluded that all studies in the sample would add to an interpretive synthesis. For example, we were aware that eight of the 24 studies were published before 2000 when quality measures in nursing research were not emphasized. Over time, quality measures have become highlighted and commonly explicated in nursing research. Inherent in qualitative designs is interpretation; we acknowledge that qualitative findings are one of many interpretations. In view of these considerations, we came to a shared understanding to include all 24 studies in the CIS, weighing them equally as we synthesized them into a conceptual understanding of responses of persons at risk for suicide.

### 3.5 Extracting data

Qualitative content analysis processes of organizing and summarizing data were used for extracting data (Granheim & Lundman, 2004). These processes involved identifying and extracting key words and concepts from results of each study. The extracted text was coded as: Disconnectedness; Grasping for connectedness; Interventions; Meaningful caring and relating; Support.

Further qualitative content analysis focused on condensing the extracted text into subthemes. Through the authors' dialogical conversations and in‐depth reflections, consensus on themes emerged. These themes were “Struggling desperately losing touch with self, others and the world; Grasping engagement releasing affliction; Pondering ways of being kept safe while moving from affliction toward the future; Contemplating meaningfulness of nurses' relating and care that fosters desire to live; Valuing support of nurses, family and systems” (Table 5). Through the unfolding and enfolding iterative and reflexive process, a CIS emerged.

**Table 5:** Results of data extraction processes

Condensed meaning units from key study findings about “Experiences of Persons at risk for suicide”	Codes	Subthemes	Themes
Patterns of suicide were: unbearable psychological pain; dissatisfactory interpersonal relationships; inability to adjust; cognitive constriction; rejection‐aggression; indirect expressions (Valente, 1994) Life stories concerned dysfunctional families of origin, poor role models, feeling isolated and being pessimistic (Haight & Hendrix, 1998) Suicide triggers were illness, pain, death of close relative or friends, family/friend/coworker conflicts/disputes and difficulty adapting to institutional life (Ku et al., 2009) Losing touch with the world involved relating suicidal attempt to life history, struggling for death and life, seeing suicide as a way to relieve desperation, feeling shame and guilt (Vatne & Nåden, 2012) Being alienated instead of connected by being controlled and rebuffed by others while wanting to leave family versus striving to live for self, seeking company of others, being loved and being responsible for family (Tzeng, 2001) Shame reactions concerned feeling failure, being ashamed of self, struggling with impulses to hide or flea and experiencing trespassing (Wicklander et al., 2003) Questioning life's meaning while experiencing psych ache and powerlessness, and perceiving nobody cared (Biong et al., 2008; Moore, 1997 Finding meaning in being isolated, being close to the point of no return, yet still being on the edge (Biong et al., 2008) Struggling to assume responsibility for self and others (searching for strength, struggling to be understood, refusing to be violated) and struggling to stay alive by enhancing self‐ development (recovering being able to be safe and trusted) (Holm & Severinsson., 2011)	Disconnected versus connected (*N* = 9)	Struggling with turbulent disconnectedness with self and others (Haight & Hendrix, 1998; Ku et al., 2009; Valente, 1994) Being alienated from self and others while striving to live (Tzeng, 2001; Vatne & Nåden, 2012) Being ashamed and feeling consumed by shame and desperation (Wicklander et al., 2003) Being perplexed about meaning in life (Biong et al., 2008; Moore, 1997) Struggling to grasp self, self‐ responsibility and self‐development (Holm & Severinsson., 2011)	Losing touch with self, others. and the world
Longing for closeness, desiring connectedness, struggling to open up inner dialogue, breaking into outer dialogue, liberating inner and outer dialogue and struggling to open up for consolation (Talseth et al., 2003) Moving from loss of support, loss of self‐esteem, loss of hope, loneliness, suffering abuse and seeking salvation to regaining hope and self‐worth (Lin et al., 2009)	Wavering to grasp connectedness (*N* = 2)	Opening up dialogue in the midst of becoming connected (Talseth et al., 2003) Pivoting from being disconnected to connected through self‐worth, safety. and hope (Lin et al., 2009)	Grasping for engagement
Overlapping stages of art future images illustrated complaint irritation, identity searching, humour reappearing, rekindling dreams, regaining control and pleasant anticipation (Walsh & Minor‐Schork, 1997) Constant observation was described as preservation of safety, restoration of hope and distressing incidents (Pitula & Cardell, 1996) Therapeutic observer interventions were: optimism, acknowledgement of the patient, use of distraction, emotional support, and protection. Nontherapeutic observer interventions were: lack of empathy, acknowledgement, information and privacy; invasion of personal space; and confinement. (Cardell & Pitula, 1999)	Interventions (*N* = 3)	Imaging a positive future through art (Walsh & Minor‐Schork, 1997) Reflecting on therapeutic and nontherapeutic ways of feeling safe and being supported in the midst of distress (Cardell & Pitula, 1999; Pitula & Cardell, 1996)	Pondering ways of being safe and connected
Care received from nurses was confirming (e.g., meeting basic needs; being seen; given time; patience, being open and nonjudgemental; conveying hope) or lack of confirming care, (i.e., unmet needs; not seen, given time or conveyed hope; and being judged) (Talseth et al., 1999) Being well cared occurred when suicidal patients received understanding and confirmation. Lack of confirmation contributed to feeling burdensome, demanding discharge and risking suicide (Samuelson et al., 2000) A key psychosocial need was “re‐connecting with humanity,” occurring through nurses “reflecting an image of humanity,” guiding one back to humanity and “learning to live.” (Cutcliffe et al., 2006) Therapeutic interpersonal engagement with nurses that helped reduce isolation, loss of control, distress and objectification of the delivery of potentially objectifying common interventions was central to quality of care (Lees et al., 2014) Encounters with healthcare personnel were identified as: the presence or absence or openness and trust; being met and not being met by someone who addressed the topic of suicide; and being met on equal terms instead of being humiliated (Vatne & Nåden, 2014) Inspiring hope though encounters with healthcare personnel within caring cultures and an atmosphere of wisdom and resuming responsibilities (Vatne & Naden, 2016b)	Meaningful caring and relating (*N* = 6)	Considering importance of confirming lack of confirming care from nurses (Samuelson et al., 2000; Talseth et al., 1999) Meaningful caring as engagement, openness, trust, and respect that re‐connects with humanity and fosters learning to live (Cutcliffe et al., 2006) Sensing being understood through the presence of caring in health personnel who actively listened and focused on meaning, inspiring hope (Lees et al., 2014; Vatne & Nåden, 2014, 2016b)	Contemplating meaningfulness of nurses' relating
Support and psychosocial needs unmet by healthcare system and nurses were being loved, esteemed, and in control of life (Carrigan, 1994) Family support affected self‐esteem, impacting suicide risk (Sharf *et al*., 2009) Recovery from suicide risk occurred through becoming aware of one's desire to live, experiencing connectedness and someone who cared (Vatne & Naden, 2016a)	Support (*N* = 3)	Desiring support from healthcare system and nurses (Carrigan, 1994) Support from family and someone who cares, the desire to live and connectedness alleviated suicide risk (Sharf et al., 2009; Vatne & Naden, 2016a)	Valuing support of nurses, family, health systems, and others

## 4 RESULTS

Relating, reflecting, translating, and weaving subthemes with themes resulted in three interpreted, synthesized concepts which describe responses of persons at risk of suicide. These concepts are: “Disengaged while fraught with affliction”; “Readiness to engage in life”; and “Engaging through caring and confirming humanity.”.

### 4.1 Concept 1. Disengaged fraught with affliction

The concept 1 emerged from the theme “Losing touch with self, others and the world.” This theme had five subthemes. The first subtheme describing persons at risk conveyed “deep struggles with turbulent disconnectedness with self and others.” Disconnectedness was portrayed as, for example, psychological pain, inability to adjust, cognitive constriction (Valente, 1994). Disconnectedness with others happened through isolation from families, conflicts with family and coworkers, poor role models, death (Haight & Hendrix, 1998; Ku, Tsai, Lin, & Lin, 2009).

The second subtheme was “being alienated from self and others while striving to live.” Alienation involved being controlled and being rebuffed by family instead of being connected to others while being caught between being responsible for family, yet responsible to strive to live for one's self (Tzeng, 2001). “Losing touch” with the world was another description that conveyed alienation (Vatne & Naden, 2012).

The third subtheme, “Being ashamed, consumed by shame and desperation,” was related to an impulse to hide or escape from shame (Wicklander, Samuelsson, & Asberg, 2003).

Amidst struggling with disconnectedness, persons at risk reflected on “being perplexed about meaning in life,” the fourth subtheme. This subtheme referred to questioning meaning. Questioning meaning related to psychache, powerlessness, and perceiving no one cared (Moore, 1997). Questioning about meaning concerned being isolated, being close to the point of no return and being on the edge (Biong & Ravndal, 2007) . The fifth subtheme, “Struggling to grasp self, self‐responsibility and self‐development,” was involved searching for strength, seeking to be understood, refusing to be violated and being responsible for one's own safety (Holm & Severinsson, 2011). This concept addresses struggling desperately to connect yet being disconnected.

### 4.2 Concept 2: Readiness to engage in dialogue

The concept 2 was synthesized from one theme and two subthemes. The theme, “Grasping for engagement,” described two subthemes. These were “Pivoting from being disconnected to connected through self‐worth, safety and hope” and “Opening up dialogue in the midst of becoming connected.” Readiness was revealed as shifting from loss of support, loss of hope, lack of self‐esteem, loneliness, abuse, searching for release, to trying to regain hope, and self‐worth (Lin, Huang, Chen, & Shao, 2009). Readiness to engage in life emerged as a process of becoming ready for dialogue. This occurred amidst longing for closeness, desiring connectedness, opening up inner and outer dialogue, and releasing dialogue (Talseth, Gilje, & Norberg, 2003).

### 4.3 Concept 3: Engaging through caring and being confirmed

The concept 3, “Engaging through caring and being confirmed,” emerged from three subthemes. The first theme, “Ponder ways of being safe and connected,” had two subthemes. The first subtheme was “Imaging a positive future through art.” The use of art connected persons at risk with their emotions, rekindled their dreams, restored their identity and regained their control, imaging the future (Walsh & Minor‐Schork, 1997). The second subtheme was “Reflecting on therapeutic and nontherapeutic ways of feeling safe and being supported in the midst of distress.” This subtheme contrasted nontherapeutic (Cardell & Pitula, 1999; Pitula & Cardell, 1996) with therapeutic (Cardell & Pitula, 1999) aspects of constant observation. Nontherapeutic aspects were lack of empathy, lack of acknowledgement, invading personal space, and confinement (Pitula & Cardell, 1996). Therapeutic aspects were optimism, being acknowledged, distracted, and providing emotional support and protection (Cardell & Pitula, 1999).

The second theme in concept 3 was “Contemplating meaningfulness of nurses” relating three subthemes which formed the basis for the emergence of this theme. The first subtheme, “Considering importance of confirming‐lack of confirming care from nurses,” contrasted the presence and absence of confirming care. Confirming care was experienced when basic needs “being met” and one was seen, given time, conveyed hope and not judged while lack of confirming care dealt with unmet needs, not being seen, not provided time, lack of hope and being judged (Talseth, Lindseth, Jacobsson, & Norberg, 1999). Being confirmed was also sensed as being understood while noncaring evoked burdensome feelings, fostering risk of suicide (Samuelson, Wiklander, Asberg, & Saveman, 2000). The second subtheme pertaining to concept 3, theme 1, was “Meaningful caring as engagement, openness, trust and respect that re‐connects with humanity and fosters learning to live.” When psychosocial needs were met, engagement reconnected one with humanity as nurses reflected an image of humanity, guiding one back to humanity while learning to live (Cutcliffe, Stevenson, Jackson, & Smith, 2006).

The third subtheme for concept 3, theme 2, was “Sensing being understood through the presence of caring in health personnel who actively listened,” focused on meaning and inspired hope. This subtheme addressed the therapeutic interaction with nurses that reduced, for example, isolation, loss of control, distress, and objectification (Lees, Proctor, & Fassett, 2014). Encounters with healthcare personnel were described as the presence or absence of openness and trust and being met or not being met by someone who acknowledged the topic of suicide and conveyed mutual respect (Vatne & Nåden, 2014). Caring encounters and caring cultures in an atmosphere of wisdom fostered resuming or assuming self‐responsibility and inspired hope (Vatne & Nåden, 2016b).

The third theme embedded in concept 3, “Valuing support from nurses, family, health system and others,” emerged from two subthemes. The first subtheme, “Desiring support from healthcare system and nurses,” involved support of psychosocial needs, being loved, and esteemed by nurses and being in control of life (Carrigan, 1994). The second subtheme was “Support from family and someone who cares, the desire to live and connectedness alleviated suicide risk.” Experiencing connectedness and someone who cared, awareness of one's desire to live (Vatne & Nåden, 2016a) along with family support, alleviated suicide risk (Sharaf, Thompson, & Walsh, 2008).

## 5 DISCUSSION

A CIS focused on understanding responses of persons at risk for suicide was conducted on research‐based nursing literature published from 1994 to May 2017 Knowledge and understanding from accumulated research‐based literature that emerged from this study increases contextual, conceptual and methodological views about this topic.

### 5.1 Contextual views

The context for most of the sample studies was Europe and North America. According to the World Health Organization (2016), the America's Region estimated suicide rates are, in general, lower than other WHO regions while the South East Asian Region has the highest estimated global suicide rate and the European Region has above the global average. However, published nursing research‐based studies from regions with high as well as low suicide rates is very sparse. Throughout history, the topic of suicide has been taboo in many areas of the world. Currently, the topics of suicide and suicidal persons are multidimensional with cultural attitudes and contexts having an impact on research.

Of importance is that much more research is needed in various contexts–geographical distributions and in variety of clinical settings. In this CIS, 18 of the 24 studies were reportedly conducted in psychiatric settings. However, suicide risks also occur in nonpsychiatric settings, including medical‐surgical units (Neville & Roan, 2013).

This study can contribute to directing and providing an impetus for future nursing research. Internationally, there are gaps in the contexts of studies addressing this topic; most have been conducted in Europe and North America. Thus, we see gaps in our global nursing knowledge. We acknowledge the challenges in various countries concerning accessing at‐risk persons as participants for sensitive research. We concur with Lakeman and Fitzgerald (2009) on the ethical and pragmatic research challenges involving persons who are suicidal. Yet, as shown in this CIS, studies have been conducted in this population. Indeed, more are needed to address sampling of global and individual cultural perspectives, ethnic diversity, age, and gender. Of note is the lack of nursing research focused on suicidal person's responses to support from, for example, family, significant others and healthcare systems. Realizing the importance of meaningful reconnections clearly points to a desire for meaningful relating and support from others and systems.

### 5.2 Conceptual views

Conceptually, this CIS contributes to a more inclusive understanding about of responses of persons at risk for suicide. The three concepts, which are not linear, but rather interwoven, can guide and direct nurses' understandings, assisting persons at risk of suicide to survive suicide risk, and go on living.

“Disengaged fraught with affliction,” reflects persons at risk desperate struggle losing touch with self, others, and the world. This is experienced as being alienated, consumed in shame, trying to grasp a sense of self and self‐responsibility while suffering with psychache. Psychache is extreme psychological pain (Sperber, 2011). Losing touch with the world alienates, experienced as “being rebuffed” by others (Tzeng, 2001), being estranged from nature, others, and self' (Sperber, 2011) and a way of “not being‐in the world” (Heidegger, 1972). Alienation encompasses loneliness and despair. It can be understood as being cut‐off from one's existence, perplexed with meaning in life. Shame is a mortifying experience involving one's own self‐evaluation of one's actions or feelings. Shame can be understood as unworthiness of the whole self (Kalafat & Lester, 2011) accompanied by a desire to flee (Tzeng, 2001; Vatne & Nåden, 2012) and extreme withdraw from the situation. Suicide becomes the ultimate withdrawal (Kalafat & Lester, 2011). When experiencing shame, we lose touch with our existence (Vatne & Naden, 2012); we fear losing the world, others and our self.

Self‐responsibility includes self‐control, being in control of life (Carrigan, 1994). For those at risk of suicide, control is about struggling with self to maintain control or to grasp regaining control (Crocker et al., 2006). Control involves being more or less connected. “Being in want of control may be a relevant and general feature of being suicidal” (2012Skogman, Bolmsjö, Edberg, & Ojehagen, 2012, p. 1). Struggling for control involves struggling for emotional balance (Berglund, Åström, & Lindgren, 2016).

“Grasping engagement releasing affliction” reveals persons at risk shifting from extreme disconnectedness to connectedness. Afflicted with shame and low self‐ worth disconnects one from self and others. As self‐worth increases and shame decreases, dialogue opens up for connecting through engagement. As engagement evolves, persons at risk begin to see themselves in the light of another person. Engagement, then, can relieve shame and foster attaching value to one's existence (c.f. Valente, 1994; Ku et al., 2009).

Feeling safe from suicidal thoughts and impulse safe in encounters with others involves “connection, protection and control,” essential to recovery from suicidal crises (Berg, Rørtveit, & Aase, 2017). It is evident that connection is important for safety. Similarly, control is important for safely. While safety includes but is more than a technical, physical intervention, of importance, safety is also about regaining emotional balance (Berglund et al., 2016) as well as engagement (c.f. Berg *et al*., 2017; Cutcliffe & Baker, 2002).

While grasping for engagement, hope wavers. Hope can waver to and fro; it can be very temporary. It needs repeating over and over while grasping connectedness (Berglund et al., 2016; Cutcliffe, 2007). Hope diminishes alienation, affirms self‐worth, fosters safety, evokes a sense of engagement, opens up for dialogue, releasing affliction.

“Engaging through receiving meaningful care and being confirmed inspires hope” is about meeting needs and being understood, esteemed and supported. These responses confirm one's humanity and inspire a desire to move into the future (Cutcliffe & Baker, 2002).

Engaging meaningfully with self and others echo a kind of “being at home—or at homeness” (Zingmark, Norberg, & Sandman, 1995). “Being at home” confirms one's humanity. Persons afflicted with suicide risk are “not at home”; they need to become ready for “being at home.” Being at home is about being in relationship, engaging meaningfully, and experiencing being confirmed. Being confirmed is a most significant aspect of life (Cissna & Sieburg, 1981, p. 259), fostering a desire to live and fostering hope. Experiencing hope gives way to strength to manage problems and can bring forth self‐control/self‐responsibility (Berglund et al., 2016).

As persons at risk engage meaningfully with nurses, they can begin to feel “at home” and self‐worth can emerge. Self‐worth emerges from experiencing confirmation. Confirmation means giving the other person the following messages: “To me, you exist! ‐ We are relating! ‐ To me, you are significant! ‐ Your way of experiencing your world is valid” (Cissna & Sieburg, 1981, p. 259). All human beings want to be confirmed for what they are and even for what they can become (Buber 1957, pp. 102–103). Making the other present means imagining what he/she perceives, feels and wishes in the moment. (Cissna & Sieburg, 1981, p. 258; Buber, 1957,. p. 102–103).

A confirming relationship inspires hope (Koehn & Cutcliffe, 2011). The desire to live, to be hopeful, is essential for those at suicide risk. Hope and caring are processes integrally woven together. These processes involve a human‐to‐human relationship, unconditional acceptance and tolerance, being heard, being understood, and feeling that one's life has value (Cutcliffe & Baker, 2002). Hope is also connected to confirming care experienced through being given time, being acknowledged, not judged yet sensing hope (Talseth et al., 1999). Similarly, being confirmed is being understood (Samuelson et al., 2000). During the processes of being cared for and being confirmed, at‐risk persons can be guided back to humanity and learn to live (Cutcliffe & Barker, 2002). From these processes, hope can emerge, revealing readiness for consolation (Talseth, Gilje & Norberg, 2003, Vatne & Nåden, 2012). Readiness for consolation emerges from opening up to move into the future. This is facilitated through examining ways for hopeful recovery. Recovery is a process that involves opening up to others to be consoled (Lakeman & Fitzgerald, 2008).

Conceptually, responses of persons at risk for suicide involve disengagement fraught with affliction, readiness to engage in life and meaningful engaging through caring and confirming humanity. Critical reflection, examining ways for a hopeful recovery, feeling cared for and confirmed in their humanity and desiring support, are ways that open up critical reflection on self, the complex meanings of life and life choices.

### 5.3 Methodological views

Methodologically, the CIS approach, applied to published studies (*N* = 24), advanced knowledge beyond aggregate data. The CIS approach had not been applied to the topic of persons at‐ risk for suicide. Dixon‐Woods et al. (2006) suggest application and evaluation of CIS in areas of health care that present challenges. Risk of suicide is a very challenging area of health care. We assert these results can be considered evidenced‐informed knowledge (c.f. Cutcliffe & Santos, 2014) and evidenced‐based nursing knowledge (c.f. Talseth & Gilje, 2011). They shift understandings to an inclusive level; they provide an understanding of accumulated nursing research‐based results about responses of persons at risk for suicide.

The CIS approach (Dixon‐Woods et al., 2006), along with qualitative content analysis (Granheim & Lundman, 2004) and systematic review of literature (Sandelowski, 2008), provided orderly ways to sort and arrange data through extraction–condensation processes. The extracted data were formulated into condensed meaning units, codes, subthemes, and themes. Reflection and clarification of the themes led to formation of three key concepts that address the aim of the CIS. We thoughtfully considered quality determinants of the data which varied yet decided on the sample of 24 studies realizing each contributed to the aggregate findings.

## 6 LIMITATIONS

As described above, the sample size was small and most studies were conducted in Europe and North America. Yet, the size was sufficient to address the research question and generate collective understandings of the topic. Of note is that suicide is a complex topic imbued with diverse cultural values and interpretations. Both authors are experienced psychiatric mental health nurse educators and qualitative researchers familiar with responses of suicidal patients. One author is from Norway and the other author is from the United States. While we view our backgrounds as both strengths and limitations to our interpretive lenses, we acknowledge the interpretations in this study are one of many (Ricoeur, 1976).

## 7 CONCLUSION

This paper presents a CIS of nursing research studies (*N* = 24) published from 1994 to 2017 on responses of persons at risk for suicide, a very vulnerable population whose safety is paramount. This understanding, based on a small sample of accumulated research‐based from nursing literature, expands contextual, conceptual, and methodological views of this topic.

Contextually, gaps are apparent in international research. Most studies were conducted in Europe and North America, and in psychiatric settings. Of note is that the context of research influences understandings of this culturally situated, sensitive topic of suicide.

Conceptually, the three key concepts (i.e., Disengaged fraught with affliction; Readiness to engage in dialogue; and Engaging through caring and being confirmed) reveals a way of understanding responses of persons at risk for suicide. These concepts can guide nurses in clinical practice as well as research.

Methodologically, the systematic review of literature and qualitative content analysis served as reasonable ways to organize data. The results can direct researchers in diverse areas of the world to further investigate responses of persons at risk for suicide. Of importance is that nurses address suicide as a preventable public health problem.

## 8 RELEVANCE TO CLINICAL PRACTICE

Many nurses will encounter vulnerable persons at risk for suicide (Lakeman, 2010). Regardless of the setting, nurses should realize that most suicidal persons can survive and go on to live. Hence, understanding ways of engaging through caring and confirming humanity can prevent suicide. Accessing and using evidenced‐informed knowledge and evidenced‐based nursing knowledge to meet the challenges of encountering these at‐risk persons, can guide nurses to facilitate hopeful recovery. Of importance is nurses' reflection on their hopefulness in working with persons at risk (Cutcliffe, 2006).

Hopeful recovery emerges from meaningful connections, caring, being confirmed as a human being. Hopeful recovery can build resilience. Nursing research on the complex unfolding processes of resilience related to depression and suicide is emerging. Depression is a known risk factor of suicide. Of note is that from 2005 to 2015, the total estimated number of people living with depression worldwide increased 18.4% (World Health Organization, 2017). Resilience is a known protective factor for depression; it is highly correlated with low depression and anxiety (Edward, 2005; Wagnild & Gantnar, 2011). Lakeman and Fitzgerald (2008) implicitly described resilience when they asserted that persons at risk for suicide can quickly turn their lives around through experiencing gaining or regaining connection with others. Hopeful recovery, a way to not give up, can potentially build resilience (Edward & Warlow, 2005). As nurses' foster resilience in those at risk for suicide, lives can turnaround and risk of suicide can be overcome, thus addressing the important work of suicide prevention in the world.

## CONFLICT OF INTEREST

The authors declare there were no conflicts of interest.

## AUTHOR CONTRIBUTIONS

Both authors substantially contributed to the conception, design, data analysis, interpretation, preparation, and refinement of the manuscript. AGT conducted the literature search which was reviewed and critiqued by both authors. FLG drafted much of the text. Both authors edited, formatted, and approved the final version of the manuscript. While our geographical residences differ, we regularly met face‐to‐face via technology and frequently via email for the study and manuscript development.

All authors have agreed on the final version and met at least one of the following criteria (recommended by the International Committee of Medical Journal Editors [https://www.icmje.org/recommendations/]):

- substantial contributions to conception and design, acquisition of data, or analysis and interpretation of data;
- drafting the article or revising it critically for important intellectual content.
